# NBZIMM: negative binomial and zero-inflated mixed models, with application to microbiome/metagenomics data analysis

**DOI:** 10.1186/s12859-020-03803-z

**Published:** 2020-10-30

**Authors:** Xinyan Zhang, Nengjun Yi

**Affiliations:** 1 Department of Statistics and Analytical Sciences, Kennesaw State University, Kennesaw, GA USA; 2 Department of Biostatistics, University of Alabama at Birmingham, Birmingham, AL 35294 USA

**Keywords:** Microbiome, Metagenomics, NBZIMM, Negative binomial mixed models, Zero-inflated mixed models

## Abstract

**Background:**

Microbiome/metagenomic data have specific characteristics, including varying total sequence reads, over-dispersion, and zero-inflation, which require tailored analytic tools. Many microbiome/metagenomic studies follow a longitudinal design to collect samples, which further complicates the analysis methods needed. A flexible and efficient R package is needed for analyzing processed multilevel or longitudinal microbiome/metagenomic data.

**Results:**

NBZIMM is a freely available R package that provides functions for setting up and fitting negative binomial mixed models, zero-inflated negative binomial mixed models, and zero-inflated Gaussian mixed models. It also provides functions to summarize the results from fitted models, both numerically and graphically. The main functions are built on top of the commonly used R packages nlme and MASS, allowing us to incorporate the well-developed analytic procedures into the framework for analyzing over-dispersed and zero-inflated count or proportion data with multilevel structures (e.g., longitudinal studies). The statistical methods and their implementations in NBZIMM particularly address the data characteristics and the complex designs in microbiome/metagenomic studies. The package is freely available from the public GitHub repository https://github.com/nyiuab/NBZIMM.

**Conclusion:**

The NBZIMM package provides useful tools for complex microbiome/metagenomics data analysis.

## Background

The recent development of technology and computational tools promotes the generation microbiome/metagenomic data, providing research opportunities to identify the links between the microbiome and diseases [1]. 16S rRNA and whole-metagenome shotgun sequencing data are two types of microbiome/metagenomic data available [2, 3]. The downstream bioinformatics pipelines will convert the raw microbiome/metagenomics sequence data into operational taxonomic units (OTUs) as count data (QIIME and mothur) or functional pathways as count data (Kraken) or proportion data (MetaPhlAn) [2, 3]. Processed microbiome/metagenomics data is over-dispersed and sparse, and has various sequencing depths, thus, it is needed to have analytic tools to address those features [4, 5]. Moreover, the number of measured taxa is usually large, thus requiring efficient algorithms for detecting significant taxa. In addition to these data features, many microbiome/metagenomic studies focused on investigated the temporal relationship of microbiome among subjects [1, 6]; for instance, they used longitudinal designs to collect multiple samples at various time points from the same subject. Thus, it is needed to account for correlation over time within and between subjects.

Various generalized linear or mixed models have been proposed to analyze the processed microbiome/metagenomic data. Negative binomial and zero-inflated negative binomial distributions are commonly used to address the over-dispersion and sparsity issues in count data, and mixed models are the standard approaches for dealing with multilevel data structures [7, 8]. Zero-inflated Gaussian distributions can be used to analyze the transformed zero-inflated count or proportion data, and zero-inflated Gaussian mixed models can handle multilevel data structures. An R package **metagenomeSeq** is available to analyze the transformed microbiome data with zero-inflated Gaussian models [9]. Meanwhile, the following R packages are available to analyze over-dispersed or sparse count data, including **pscl**, **mgcv**, **brms**, **gamlss**, **GLMMadaptive**, and **glmmTMB** [10–14]. The **pscl** package is a popular package in fitting zero-inflated and hurdle model but cannot handle repeated measures or longitudinal studies. The package **mgcv** can fit negative binomial mixed models (NBMMs), but the value for the dispersion parameter in NBMMs needs to be specified. Also, NBMMs fitted by **mgcv** can only include simple random effects without the options to handle within-subject residual correlation structures [7]. Besides, using **mgcv**, we can use zero-inflated Poisson mixed models to analyze sparse data but may not be appropriate to model the sparse over-dispersed count data. The other R packages can set up both NBMMs and zero-inflated negative binomial mixed models (ZINBMMs) but may not be ideal in analyzing microbiome/metagenomics data due to computational efficiency. The **brms** package fits the models using MCMC sampling, which is the slowest algorithm among the four packages. The package **gamlss** uses a Newton–Raphson or Fisher-scoring based algorithms [12]. But with the presence of random effects, the standard deviations for fixed effects fitted by **gamlss** are largely under-estimated. **GLMMadaptive** and **glmmTMB** use adaptive Gaussian quadrature and Laplace approximation, respectively, to fit ZINBMMs. Moreover, none of the above-mentioned R packages are designed to analyze microbiome/metagenomic data, thus we cannot directly summarize or visualize the analysis results of many taxa or functional units with those R packages. Alternatively, Chen and Li [15] proposed a zero-inflated Beta regression model with random effects (ZIBR) for analyzing longitudinal microbiome proportion data, which is implemented in the R package **ZIBR** [16]. However, **ZIBR** requires that the number of time points be the same for various subjects.

A flexible and efficient R package is needed for analyzing processed multilevel or longitudinal microbiome/metagenomic data. Here, we introduce the freely available R package **NBZIMM** for analyzing processed complex microbiome/metagenomic data, which implements our published methods [5, 17, 18]. In our previous work, we have evaluated and compared the three methods NBMMs, ZINBMMs and zero-inflated Gaussian (linear) mixed models (ZIGMMs) implemented in **NBZIMM** with various existing methods in different R packages. We found that ZINBMMs in **NBZIMM** is improved in computational time but was comparable in statistical analysis with two other R packages **GLMMadaptive** and **glmmTMB** to fit ZINBMMs [10, 11, 18]. We also found that ZINBMMs outperform NBMMs and ZIGMMs in power for sparse data but may be similar to the other methods in not highly sparse microbiome data through simulation studies [18]. We also have evaluated the methods in **NBZIMM** in public available datasets and found that the taxon identified by NBMMs and ZINBMMs are mostly either overlapped with the original paper or have been reported in previous research [5, 17, 18]. **NBZIMM** provides three main functions to fit NBMMs, ZINBMMs, and ZIGMMs. The main difference between **NBZIMM** and some other aforementioned R packages is the model-fitting algorithm. The algorithms used to fit NBMMs, ZINBMMs, and ZIGMMs in **NBZIMM** are all fast and stable [5, 17, 18]. It also has functions for numerically or graphically summarizing the results. The main functions are developed based on the commonly used R packages, **MASS**, and **nlme**, for analyzing negative binomial models and linear mixed models (LMMs) [7, 8], and inherit powerful features of the standard tools. The main functions are flexible to include various forms of fixed and random effects and allow the specification of within-subject residual correlation structures [7]. Here, we are presenting **NBZIMM** as an efficient and flexible tool and providing a detailed demonstration of how to use **NBZIMM** in analyzing processed longitudinal microbiome/metagenomic data.

## Implementations

We describe our models and algorithms with a two-level design where samples are grouped in subjects. Assume that a microbiome/metagenomic study collects *n* subjects and *n*_*i*_ samples for the *i*-th subject. For each sample, we measure the counts for *m* taxa (OTU, species, genus, etc.), *C*_*ijh*_; *h* = 1, ···, *m*, the total sequence read *T*_*ij*_, and some relevant covariates *X*_*ij*_. The goal is to identify if there is any microbiota taxa could be lined with covariates of interest in a study.

The function *glmm.nb* in **NBZIMM** allows us to analyze the data for taxon *h* with NBMMs:

1 $$C_{ijh} \sim {\text{NB}}(C_{ijh} |\mu_{ijh} ,\theta_{h} ), \, \log \mu_{ijh} = \log (T_{ij} ) + X_{ij} \beta_{h} + G_{ij} b_{ih} , \, b_{ih} \sim N(0,\Psi_{h} )$$

where the dispersion *θ*_*h*_ determines the over-dispersion, the offset log(*T*_*ij*_) accounts for the varying total sequence reads, *β*_*h*_ is a vector of fixed effects, and *b*_*ih*_ are random effects. *G*_*ij*_ denotes the vector of group-level covariates. *glmm.nb* iteratively approximates the NBMMs by fitting a linear mixed model using the function *lme* from the package **nlme**. The dispersion *θ*_*h*_ is then updated using Newton–Raphson algorithm as in the function *glm.nb* of **MASS**. This framework allows us to incorporate the powerful features of *lme* into NBMMs.

The function *glmm.zinb* implements ZINBMMs that directly model true zeros and can be more efficient for analyzing taxa with excessive zeros than *glmm.nb*:

2 $$C_{ijh} \sim \left\{ {\begin{array}{*{20}l} 0 \hfill & {{\text{with}}\;{\text{probability}}\;p_{ijh} } \hfill \\ {{\text{NB}}(C_{ijh} |\mu_{ijh} ,\theta_{h} )} \hfill & {{\text{with}}\;{\text{probability}}\;1 - p_{ijh} } \hfill \\ \end{array} } \right.$$

Here, the means *μ*_*ijh*_ are modeled as above, and the zero-inflation probabilities *p*_*ijh*_ are assumed to depend on some covariates via a logistic regression logit(*p*_*ijh*_) = *Z*_*ij*_*α*_*h*_ or logistic mixed model logit(*p*_*ijh*_) = *Z*_*ij*_*α*_*h*_ + *G*_*ij*_*a*_*ih*_, where *Z*_*ij*_ denotes the potential covariates associated with the excess zeros, *α*_*h*_ is a vector of fixed effects and the random effects *a*_*ih*_ ~ N(0, Φ_*h*_). To fit the ZINBMMs, *glmm.zinb* uses an efficient and stable EM-IWLS algorithm, making use of the function *lme *from the package **nlme**.

Rather than directly analyzing the observed counts, some methods analyze transformed count data [9], for example, *y*_*ijh*_ = log_2_(*C*_*ijh*_ + 1). Also, in some bioinformatics pipeline, such as MetaPhlAn, the whole metagenome shotgun sequencing data are processed and output in terms of relative abundance as proportion data. The transformation for proportion data is commonly chosen as arcsine square root transformation $y_{{ijh}} = {\text{arcsine}}\left( {\sqrt {C_{{ijh}} /T_{{ij}} } } \right)$. The function *lme.zig* in **NBZIMM** implements ZIGMMs for the transformed count or proportion data:

3 $$y_{{ijh}} \sim \left\{ {\begin{array}{ll} 0 \hfill & {{\text{with}}\;{\text{probability}}\;p_{{ij}} } \hfill \\ {N(y_{{ijh}} |\mu _{{ijh}} ,\sigma ^{2} )} \hfill & {{\text{with}}\;{\text{probability}}\;1 - p_{{ij}} } \hfill \\ \end{array} } \right.$$

where *μ*_*ijh*_ denotes the means and *p*_*ijh*_ are the zero-inflation probabilities. The function *lme.zig* uses an efficient EM algorithm to fit ZIGMMs.

The data inputs and specifications of fixed and random effects in the above functions are similar to *lme*, allowing us to incorporate all the forms of fixed and random effects implemented in *lme* into our models. In the functions *glmm.*zinb and lme.zig, there are two options to include various forms of fixed and random effects in the zero-inflated part. The function *lme* can specify various forms of within-subject residual correlation structures [7], for instance, AR(1), which also have been incorporated into our framework. Moreover, the models fitted by our main functions, *glmm.nb*, *glmm.zinb*, and *lme.zig*, can be directly fed to the function *summary* from **nlme**, which returns the estimates, standard deviations and *p* values of fixed effects, and the estimates of the variances of random effects, etc. These features made **NBZIMM** easy to use, flexible and comprehensive in modeling and stable and efficient in computation.

Although the above three functions only model one taxon at a time, **NBZIMM** includes a wrapper function *mms* to screen all included taxa, by repeated calling to *glmm.nb*, *glmm.zinb*, *lme*, or *lme.zig*. The function *fixed* extracts the estimates, standard deviations and *p* values for fixed effects of all the taxa and covariates, while *get.fixed* extracts the estimates, standard deviations and *p* values of fixed effects for a given taxon or covariate. The **NBZIMM** package provides two functions to graphically display the analytic results from *mms*. The function *plot.fixed* plots the estimates, intervals, and *p* values for numerous fixed effects. It uses different colors to distinguish between significant and insignificant effects. The function *heat.p* displays **ggplot2**-based heat map to visualize *p* values and positive or negative signs of significant effects for numerous taxa and multiple covariates.

## Results

In the recent published microbiome studies [19, 20], both sequence data and processed abundance data tables were made available. **NBZIMM** is only capable to analyze the processed abundance data tables. In this section, we will demonstrate the use of NBMMs, ZINBMMs, and ZIGMMs in analyzing processed longitudinal microbiome/metagenomics data. The example datasets in the demonstrations are consisted of three components: clinical data, taxa abundance data table (such as OTU microbiome data) and taxonomy table. The taxa abundance data that **NBZIMM** could analyze are processed abundance data table such as the file in the HMP DACC (https://www.hmpdacc.org/hmsmcp2/) [19].

### Demonstrations of NBMMs and ZINBMMs for directly analyzing longitudinal microbiome/metagenomics count data

NBMMs could analyze over-dispersed longitudinal microbiome/metagenomics count data. We will describe the functions *glmm.nb* and *mms* and show how to set up the NBMMs with those two functions. The functions *glmm.nb* and *mms* work by calling the function *lme* from package **nlme** repeatedly to fit LMMs, so both functions have the same options to include different forms of random effects and within-subject correlation structures as in the function *lme*.

To address the sparsity issue in longitudinal microbiome/metagenomics count data, ZINBMMs is also available to analyze over-dispersed and zero-inflated longitudinal microbiome/metagenomic count data in **NBZIMM**. The functions *glmm.zinb* and *mms* in **NBZIMM** are created to fit the ZINBMMs. The function *glmm.zinb* and *mms* call to the function *lme* from the package **nlme** repeatedly to fit the weighted linear mixed model. And they also call *glm* in the package **stats** or *glmPQL* in **MASS** to fit the logistic regression or logistic mixed model. Similar to the function *glmm.nb*, the specification of random effects and within-subject correlation structures in *glmm.zinb* and *mms* are the same as described in the function *lme*.

Here, we demonstrate the use of *glmm.nb*, *glmm.zinb*, and *mms* to analyze an example data with NBMMs and ZINBMMs. The microbiome data is publicly available from a case–control longitudinal study, which is designed to explore the differences of the vaginal microbiota composition between pregnant and non-pregnant women [6]. This study included 22 pregnant women who delivered at term and 32 non-pregnant women and collected samples at multiple time points. The data consist of two data components: OTU and Clinical Data. OTU contains microbiome count data for 900 samples with 143 taxa; Clinical Data contains data of clinical variables for all the samples with 9 variables, including subject ID, pregnant status, total sequencing read, age, race, etc. In the R package, Clinical Data is saved in the data component with the name of SampleData.
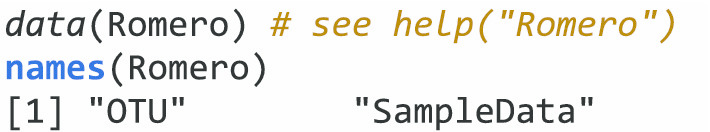


In the SampleData component, there are 9 clinical variables; variable *Subject_ID* is the subject ID, variable *pregnant* represents pregnant status, variable *Total.Read.Counts* are the total sequencing read, variable *GA_Days* indicates the time of sample collection, and there are two other covariates age and race included in the dataset.
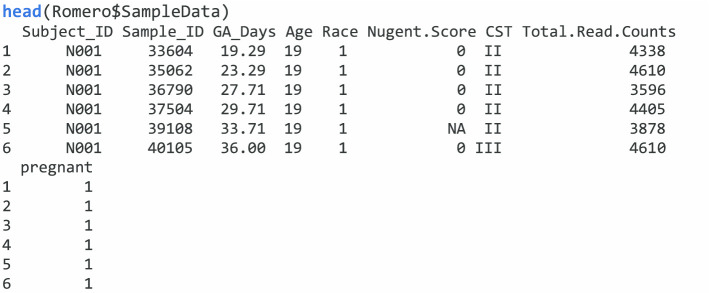


The function nonzero calculates the proportion of non-zero values for each response and plots the proportions.
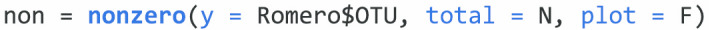


#### Analysis of a single taxon each time

We first show the analysis of a single taxon (Lactobacillus) with the function *glmm.nb* and *glmm.zinb*, respectively. The case–control group variable is defined as pregnancy women vs non-pregnant women. We consider the sample collection time as the time variable and also include covariates, age, and race. The main research goal is to explore the association between the bacterial taxonomic composition for the vaginal microbiota and the group variable of interest. Different models can be used to explore the association. The following codes are to run two examples of using a random intercept model by assuming negative binomial or zero-inflated negative binomial distributions. An offset term is needed to adjust for the library size *N* when analyzing a longitudinal microbiome count data. By assuming negative binomial distribution for the OTU, we can model it with *glmm.nb*:
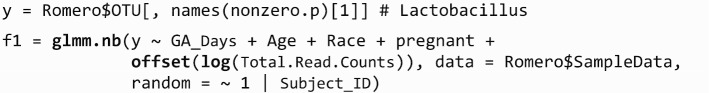


Or assuming zero-inflated negative binomial distribution, we can model it with *glmm.zinb*.
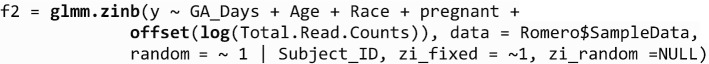


In both functions, we have a *fixed* term to include the formula for the fixed-effects part to specify the outcome and various predictors. We also provide a *random* term to include the formula for the random-effects part. It only contain the right-hand side part, e.g., ~ 1 | subject. The *data* term is to include a data frame with all the variables. In function *glmm.zinb*, there are two terms *zi_fixed* and *zi_random*, which are to specify the formula for the fixed and random effects of the zero-inflated part. The two terms, *zi_fixed* and *zi_random*, both only contain the right-hand side part. The following codes show two examples of using NBMMs and ZINBMMs to analyze the single taxon (Lactobacillus). The first example is a random slope model with NBMMs. The second example is a random slope model while controlling a covariate in the fixed effects of zero-inflated part using ZINBMMs.
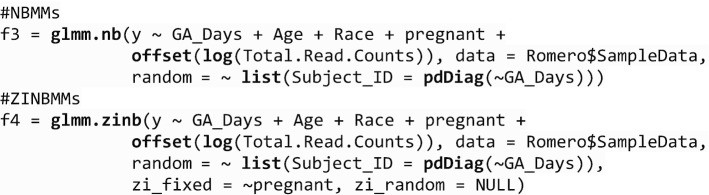


We can also incorporate autoregressive of order 1, AR(1) for correlation matrix ${R}_{i}$ to describe dependence among observations in the *correlation* term. The correlation matrix is not restricted to be AR(1) but can take any form that is available in *corClasses* from **nlme**.
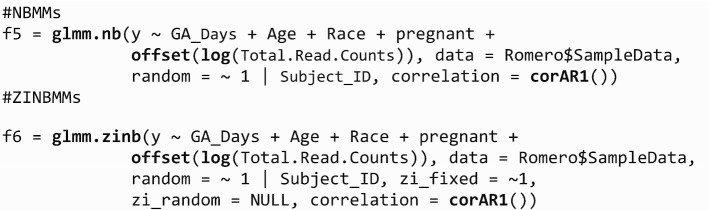


To summarize the results we use *summary* function.
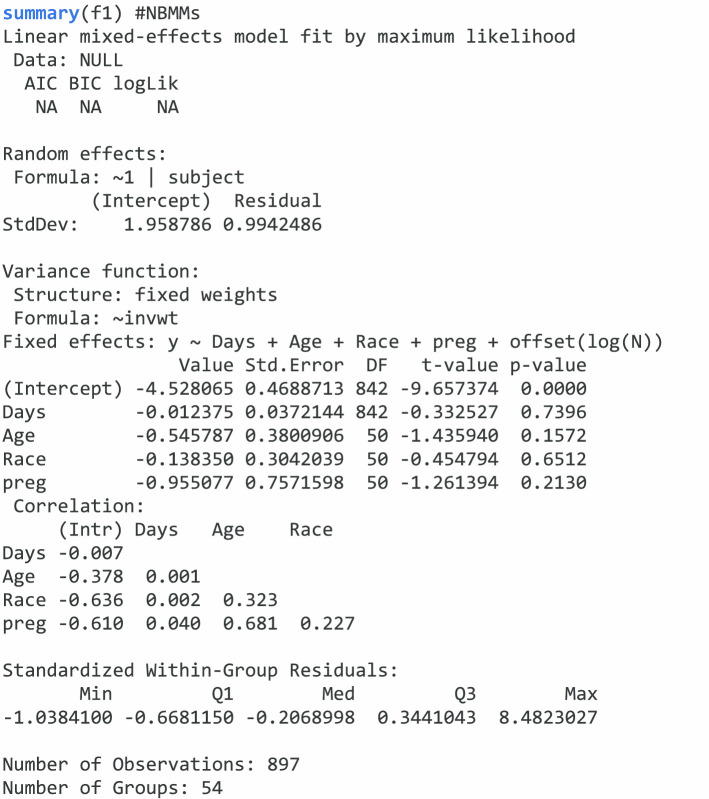


#### Analysis of all taxa with a given nonzero proportion with function *mms*

For the microbiome/metagenomics data, it is more common that we have many taxa of interest, it is easier to analyze them all at one time with the function *mms*. For both NBMMs and ZINBMMs, the function *mms* can analyze multiple taxa at one time. In this function, *y* term is to include a matrix of responses for all taxa of interest; the *fixed* term here is to set a one-sided formula of the form ~ x (i.e., the response is omitted); the right side of ~ is the same as in *glmm.nb*, or *glmm.zinb*.

The terms *data*, *random*, *correlation*, *zi_fixed*, and *zi_random *are the same as in the functions *glmm.nb*, or *glmm.zinb*. The term *Method* is to specify the distribution to be assumed for the response data, such as “nb” for negative binomial or “zinb” for zero-inflated negative binomial. The term *sort* is to sort by the nonzero proportions of the responses into decreasing order. The term *min.p* is to set an inclusion criteria to analyze taxa with a given nonzero proportion. The following example analyze all the taxa with the proportion of non-zero values > 0.2 through the term *min.p* with NBMMs and ZINBMMs.
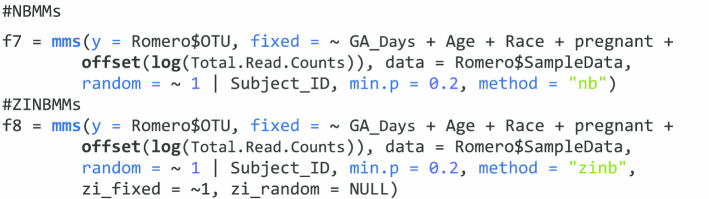


#### Visualize the results

**Fig. 1:**
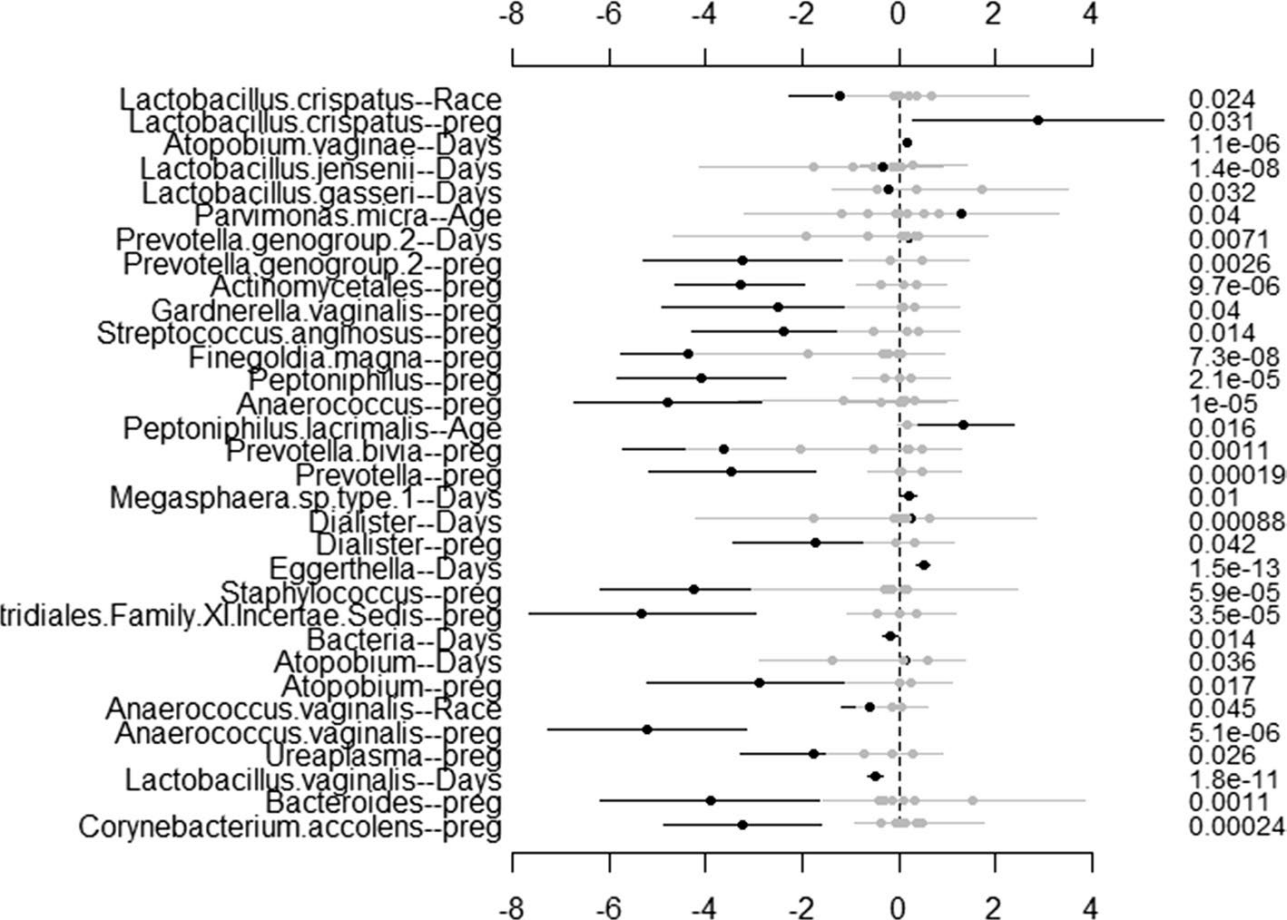
Effects between significant taxa and all covariates for Romero et al. [6] using NBMMs. The left labels the taxa name and corresponding covariate. The right labels the *p* value

**Fig. 2:**
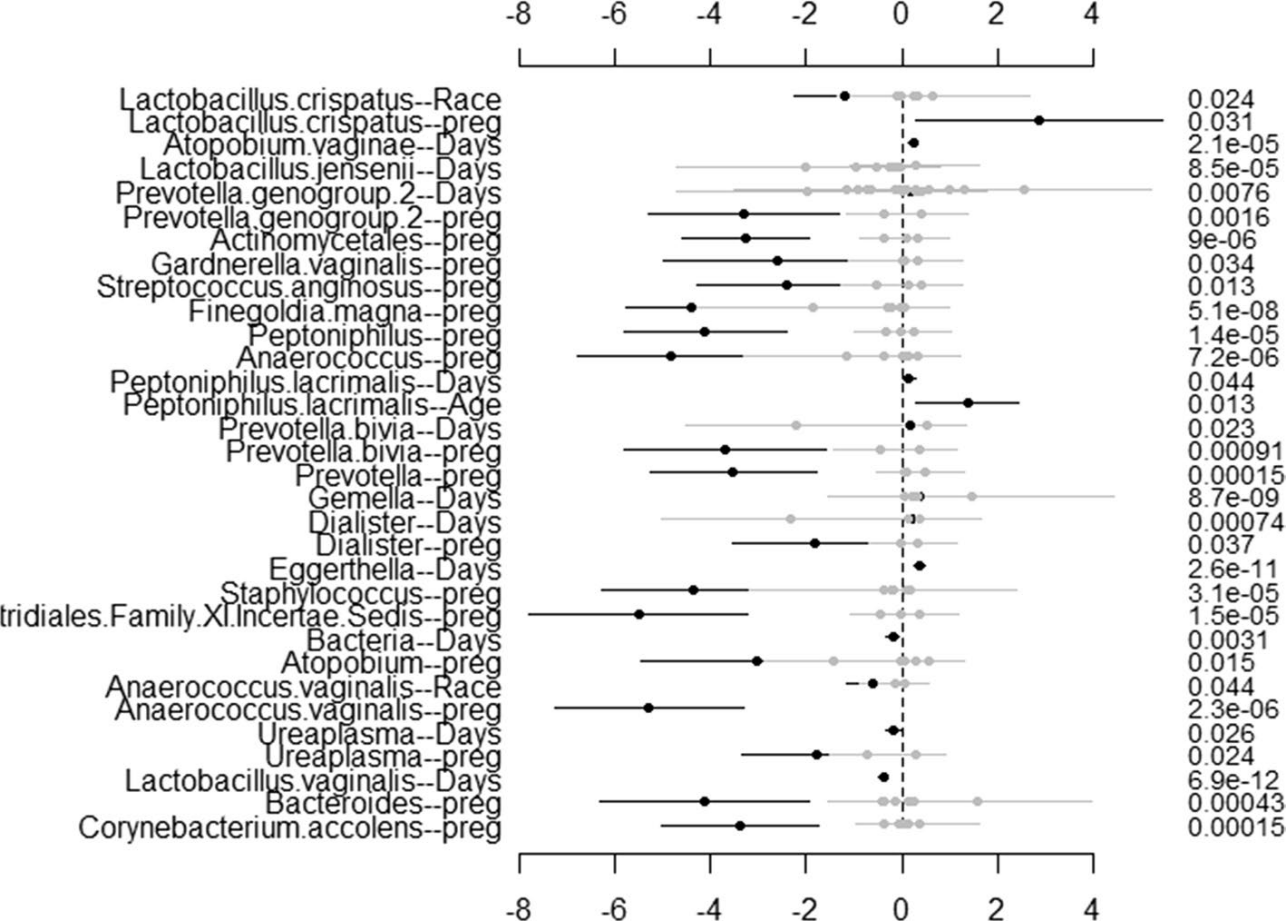
Effects between significant taxa and all covariates for Romero et al. [6] using ZINBMMs. The left labels the taxa name and corresponding covariate. The right labels the *p* value


To visualize the results through **NBZIMM**, there are several options available in the package. First, we can generate a plot with function *plot.fixed* to view the significant taxa associated with various covariates and corresponding *p* values. We will show the example plots for both NBMMs and ZINBMMs in Figs. 1 and 2. And comparing Figs. 1 and 2 in analyzing the taxa from Romero et al. [6], the results from NBMMs and ZINBMMs are similar with slight differences in *p* values.

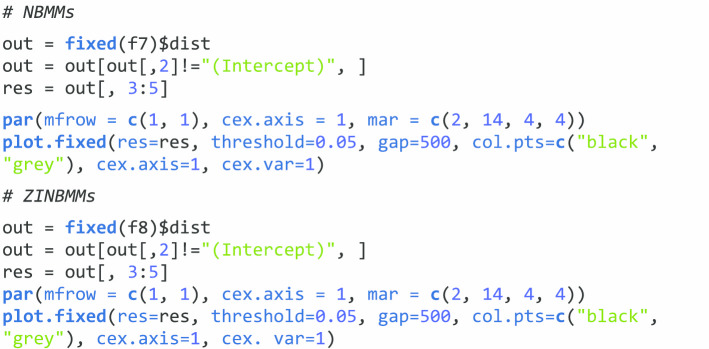


**Fig. 3:**
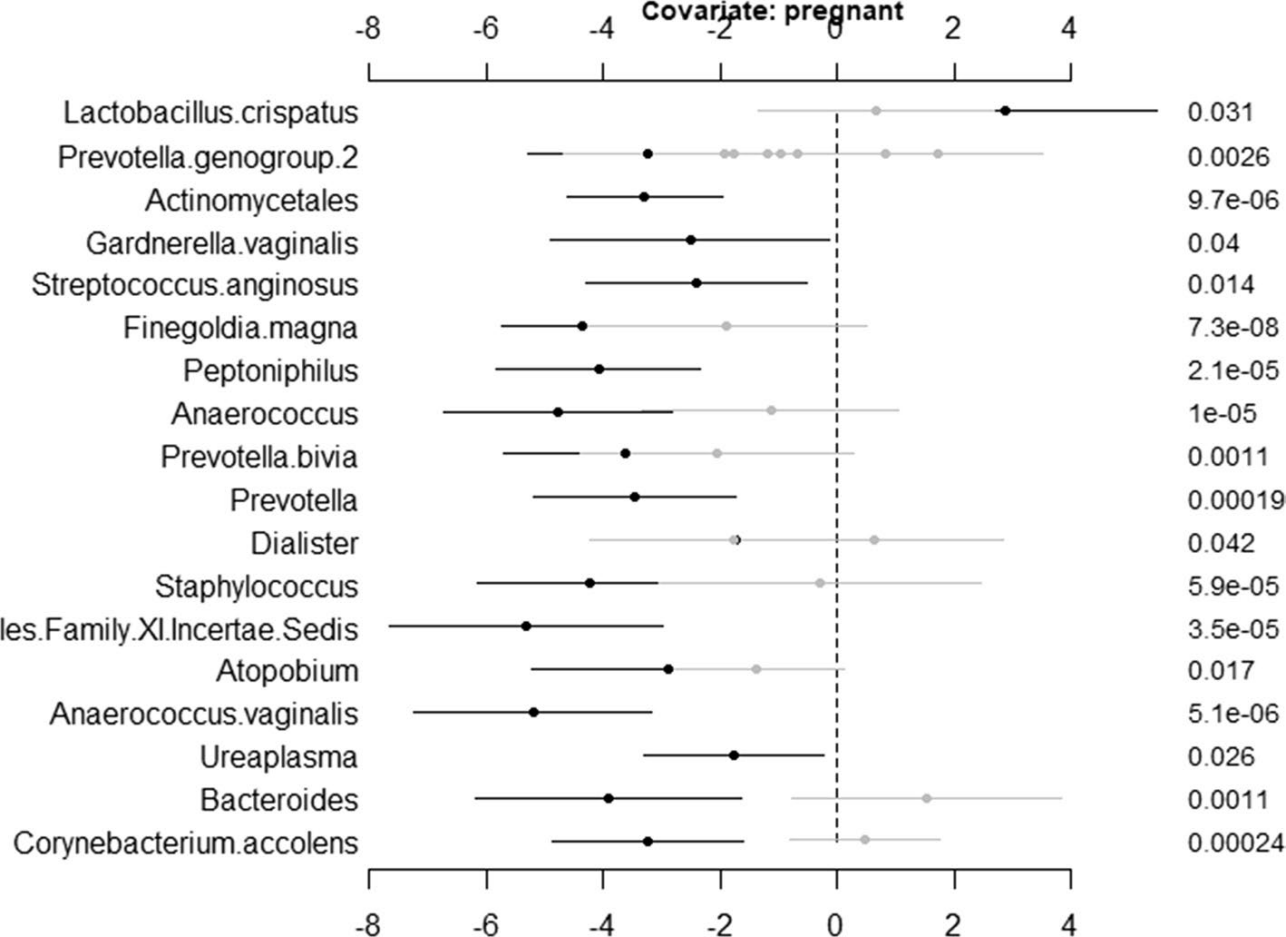
Effects between significant taxa and covariate Pregnant for Romero et al. [6] using NBMMs. The left labels the taxa name and corresponding covariate. The right labels the *p* value


The second option is that we can choose only to display the significant taxa specially associated with a covariate of interest. To generate this plot, the object *res* needs to be updated with the function *get.fixed* first and then plotted using the function *plot.fixed*. The following code is to generate Fig. 3 to visualize the associations between the variable pregnant and the taxa from Romero et al. [6] using NBMMs.

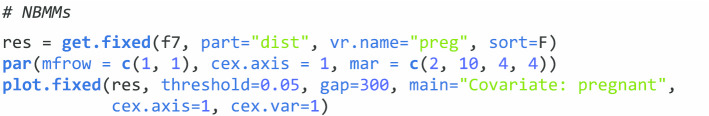


**Fig. 4:**
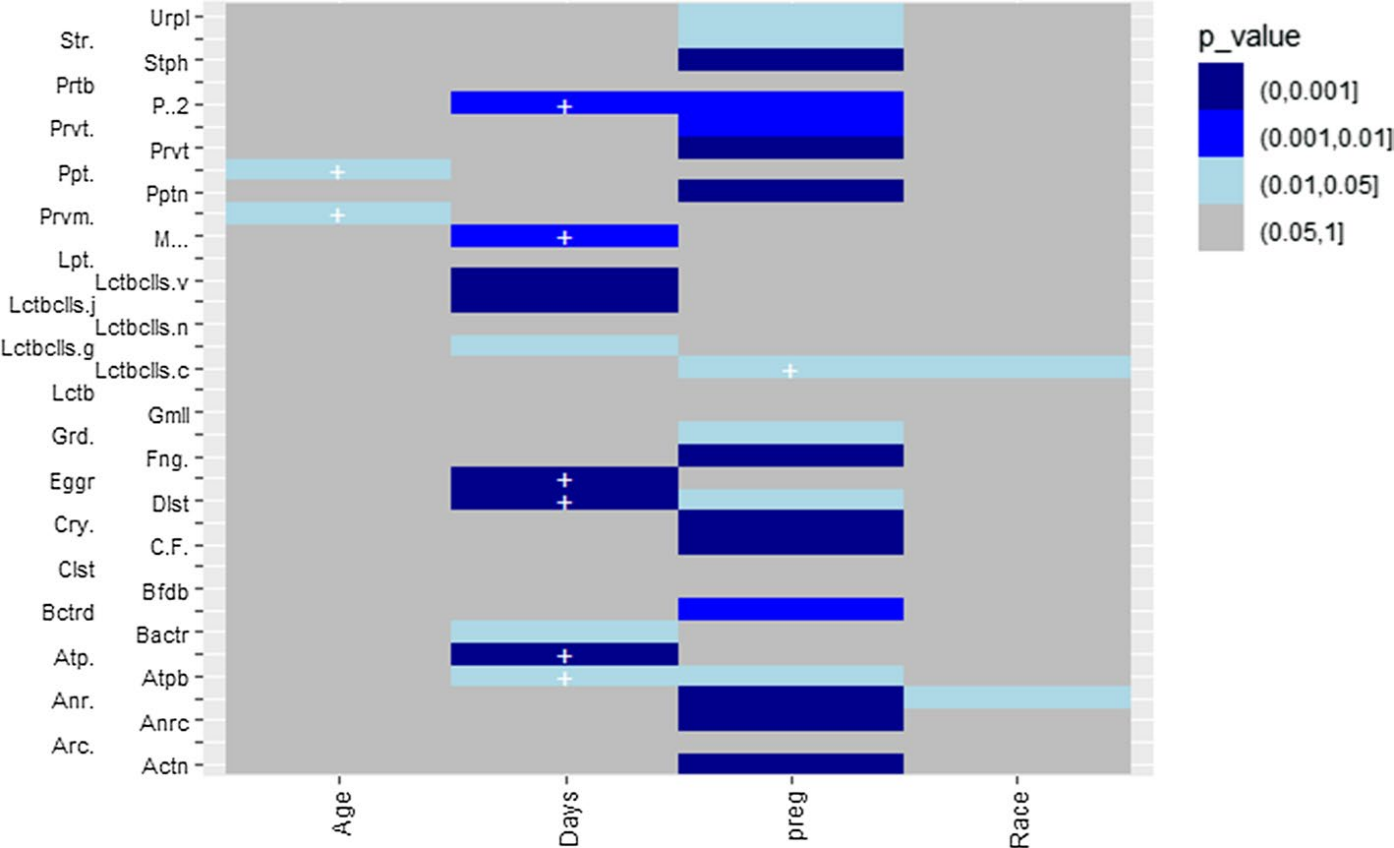
A heat map for *p* values from analyzing Romero et al. [6] with NBMMs. The taxa on y-axis are abbreviated. The sign "+" indicates a positive effect


Moreover, another option is available to generate a heat map to present the results through the function *heat.p*. The following code is to generate a heat map to visualize the associations between all four covariates and the taxa from Romero et al. [6] using NBMMs in Fig. 4.

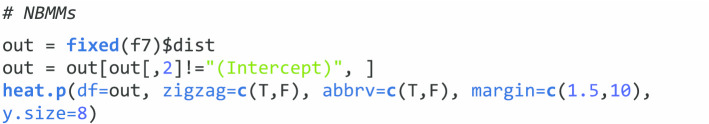


### Demonstrations of ZIGMMs in analyzing longitudinal microbiome/metagenomics count data

Longitudinal microbiome/metagenomics count data can be analyzed with ZIGMMs in **NBZIMM** through the functions *lme.zig* and *mms*. For count data, ZIGMMs analyze the count data with the transformation log_2_(*C*_*ijh*_ + 1). The function *lme.zig* calls the function *lme *repeatedly from the R package **nlme** and *glm* or *glmPQL* from the package **MASS**. *lme* fits the weighted linear mixed model; and *glm* or *glmPQL* fits the binomial logistic or mixed logistic model. We demonstrate the use of ZIGMMs in analyzing the same public microbiome count data from Romero et al. [6].

#### Analysis of a single taxon each time

We first show the analysis of a single taxon (Lactobacillus) with the function *lme.zig*. The group variable of interest and covariates are the same as mentioned in “Demonstrations of NBMMs and ZINBMMs for directly analyzing longitudinal microbiome/metagenomics count data”. section The following code is an example of analyzing a single taxon with ZIGMMs using a random intercept. An offset term is needed to adjust for the library size *N* when analyzing transformed longitudinal microbiome count data.
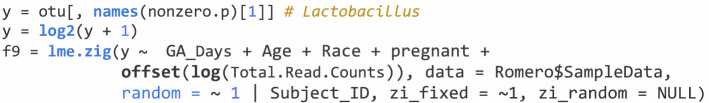


In function *lme.zig*, we also have *fixed* and *random* terms to include the formula for the fixed-effects and random-effects parts. The *data* term is to include a data frame with all the variables. Also, in this function, we can add fixed and random effects to the zero-inflated part for ZIGMMs through terms *zi_fixed* and *zi_random*. We can incorporate any correlation matrix ${R}_{i}$ available in *corClasses* from **nlme** to describe dependence among observations through the *correlation* term. The following codes show two examples of using ZIGMMs to analyze a single taxon. The first example is random slope model incorporated a correlation structure AR(1). The second example is a random intercept model while controlling a covariate in the fixed effects of zero-inflated part.
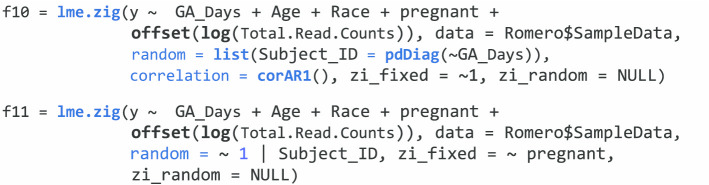


Similar as *glmm.nb* and *glmm.zinb*, we can also summarize the results using *summary* function for *lme.zig*.
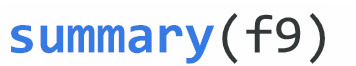


#### Analysis of all taxa with a given nonzero proportion with function *mms*

Similarly, for many taxa of interest, we analyze them all at one time with the function *mms*. The term *Method* needs to be set as ‘zig’ for ZIGMMs. The following example analyzes all the taxa with the proportion of non-zero values > 0.2 through the term *min.p* using ZIGMMs.
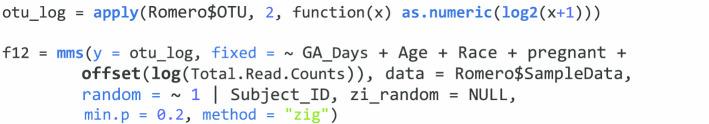


Besides, for visualization of the results, the options are the same as for NBMMs and ZINBMMs.

### Demonstrations of ZIGMMs in analyzing longitudinal microbiome/metagenomics proportion data

ZIGMMs is also applicable in analyzing longitudinal microbiome/metagenomics proportion data with arcsine square root transformation through the functions *lme.zig* and *mms*. We will demonstrate the use of ZIGMMs in analyzing longitudinal microbiome/metagenomics proportion data through the following public data demonstration. This data demonstration used a published dataset from Vincent et al. [21]. Vincent et al. [21] collected fecal samples from 98 subjects and used whole metagenome shotgun sequencing to examine the composition of their fecal microbiota. The prospective cohort study was carried out among 8 patients who were Clostridium difficile infected or colonized (CDI) and other 90 patients. The clinical covariates in the dataset are gender, age, and days from first collection of the fecal samples. The dataset was downloaded with R package curatedMetagenomicData, which contains clinical data and metagenomic data [22]. The metagenomic data is in the form of proportion data. So, the proportion data will be transformed with arcsine square root transformation to be analyzed by ZIGMMs. First, we will load the data object “vincent2016.RData” into R and check the clinical covariates. The data object includes two parts, ‘clinical’ and ‘out’. There are 4 clinical covariates included in the data object ‘clinical’; variable s*ubjectID* is the subject ID, variable *days_from_first_collection* indicates the time of sample collection, variable *number_reads* is the total sequencing read, and there are two other covariates age and gender included in the data object ‘clinical’. Also, we generated a grouping variable for patients with CDI vs controls and scaled the matrix of metagenomics data by 100 to relative proportions.
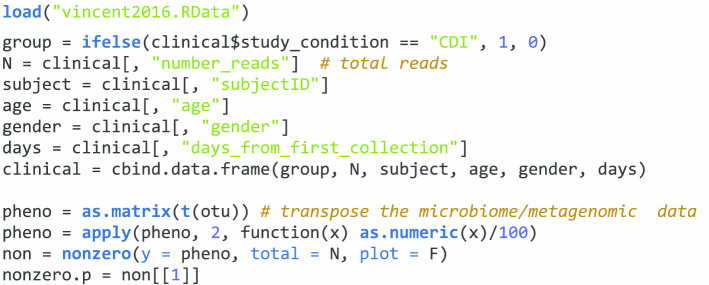


#### Analysis of a single taxon each time

We first show the analysis of a single taxon (Prevotella Bivia) with the function *lme.zig*. We are interested to compare CDI with healthy controls, which defines the group variable of interest in this example. We consider the sample collection time as the time variable and also include covariates, age, and gender. The main research goal is to explore the diversity and composition of the fecal microbiota between CDI vs healthy controls. Different models can also be used to explore the relationship. The following code is an example of a random intercept model using ZIGMMs. An offset term should not be included as we are analyzing proportion data. In function *lme.zig*, we have described the terms *fixed*, *random*, *data*, *zi_fixed*, *zi_random*, and *correlation* in “Demonstrations of ZIGMMs in analyzing longitudinal microbiome/metagenomics count data” section. By assuming zero-inflated Gaussian distribution for the transformed relative abundance proportion data, we can model it with *lme.zig* as:
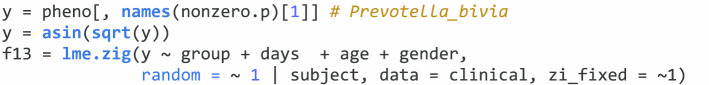


The following codes show another two examples of using ZIGMMs to analyze the relative abundance proportion for a single taxon. The first example is random slope model incorporated a correlation structure AR(1). The second example is a random intercept model while controlling the group variable in the fixed effects of zero-inflated part. We can then also summarize the results using *summary* function for the results generated by *lme.zig*.
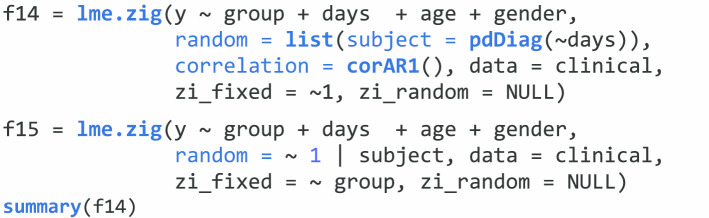


#### Analysis of all taxa with a given nonzero proportion with function *mms*

Similar as mentioned in “Demonstrations of NBMMs and ZINBMMs for directly analyzing longitudinal microbiome/metagenomics count data” and “Demonstrations of ZIGMMs in analyzing longitudinal microbiome/metagenomics count data” sections, for many taxa of interest, we can also analyze their relative abundance proportion data all at one time using the function *mms*. To use ZIGMMs, the term *Method* needs to be set as ‘zig’ for ZIGMMs. Here we use the following example to show how to analyze the transformed relative abundance proportion data for the first 30 taxa using ZIGMMs. And we used the term *min.p* to control that only the taxa with the proportion of non-zero values > 0.2 to be analyzed.
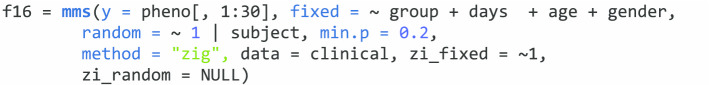


#### Visualize the results

**Fig. 5:**
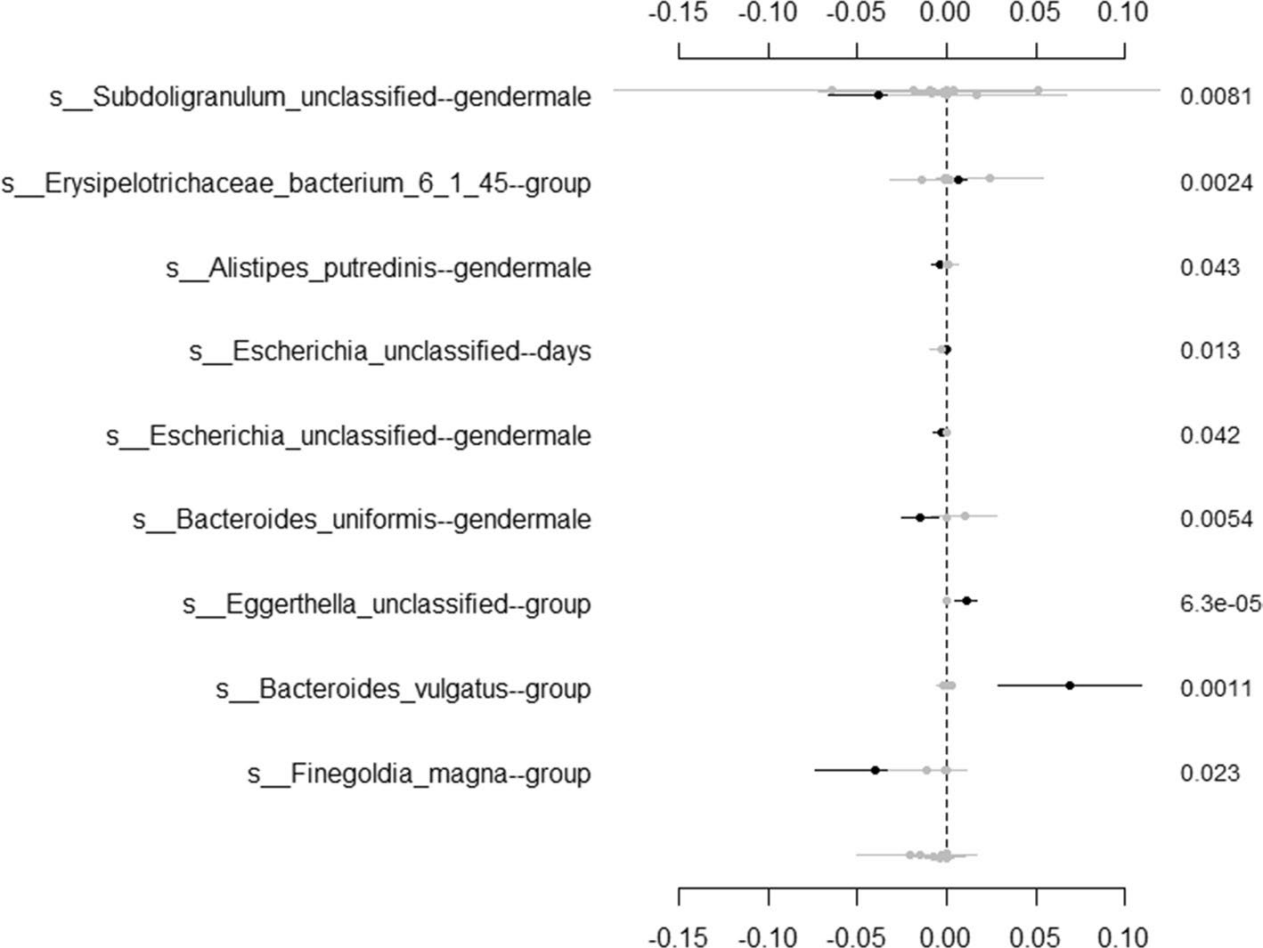
Effects between significant taxa and all covariates for Vincent et al. [21] using ZIGMMs. The left labels the taxa name and corresponding covariate. The right labels the *p* value

**Fig. 6:**
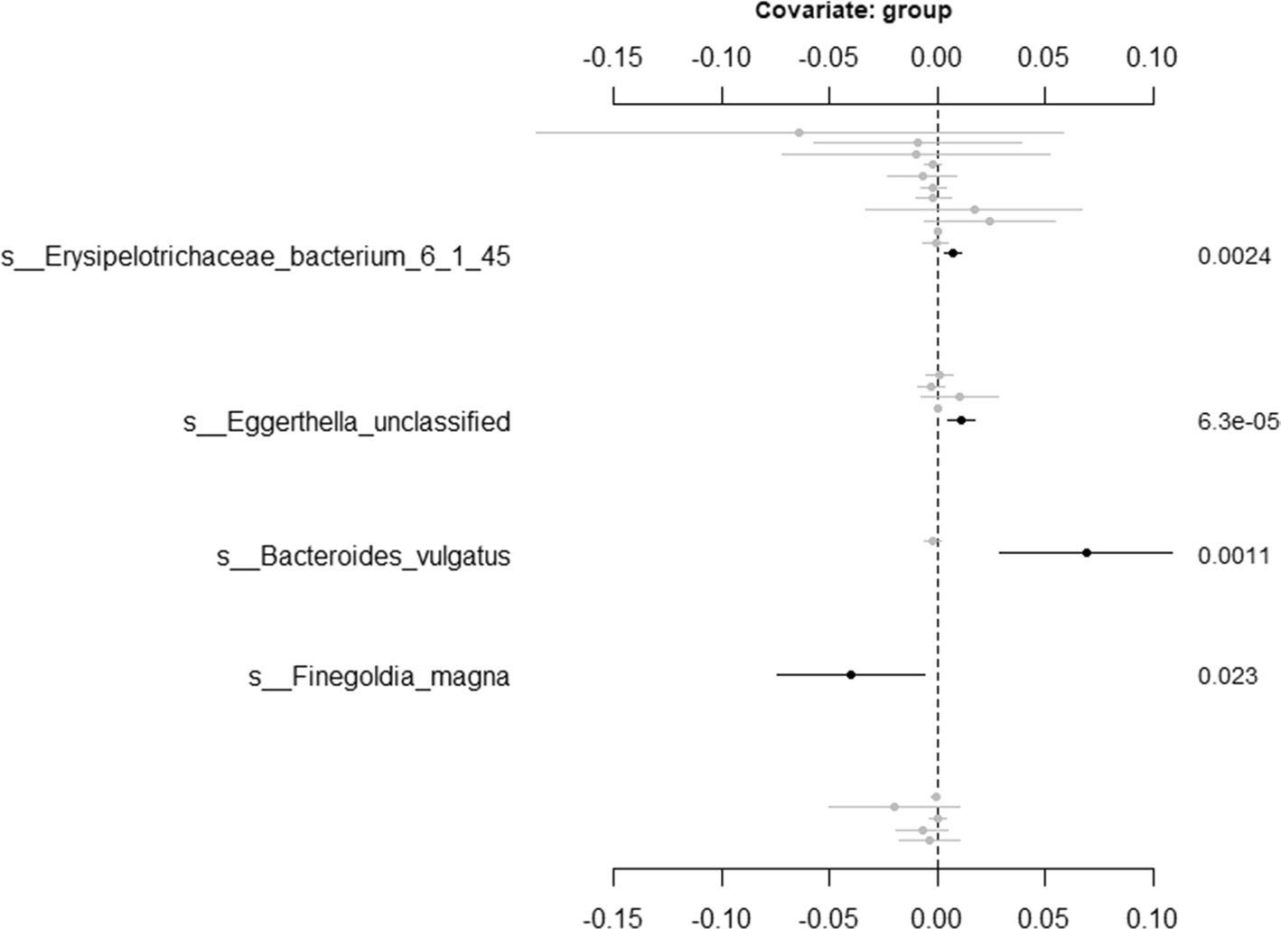
Effects between significant taxa and covariate group for Vincent et al. [21] using ZIGMMs. The left labels the taxa name and corresponding covariate. The right labels the *p* value

**Fig. 7:**
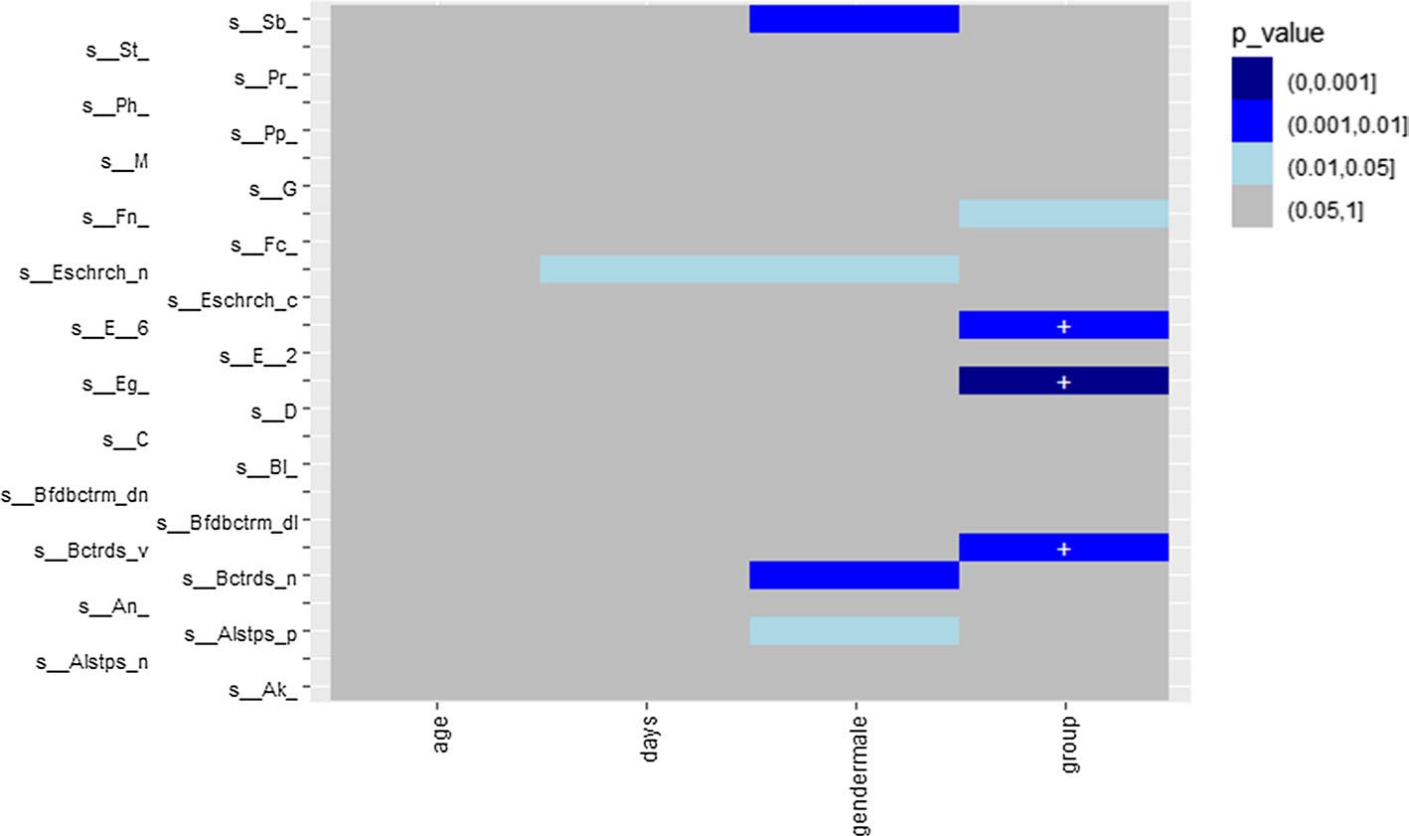
A heat map for *p* values fitted with ZIGMMs for Vincent et al. [21]. The taxa on y-axis are abbreviated. The sign “+” indicates a positive effect


To visualize the results, we have the same three options as mentioned in “Demonstrations of NBMMs and ZINBMMs for directly analyzing longitudinal microbiome/metagenomics count data” section. Figure 5 is generated by *plot.fixed* to view the significant taxa associated with various covariates and corresponding *p* values. We choose only to check the significant taxa especially associated with the variable group shown in Fig. 6. We first updated the object *res* with the function *get.fixed*, setting the term *vr.name* as group. We then plotted Fig. 6 using the function *plot.fixed*. Another option is to generate a heat map as an example shown in Fig. 7 using the function *heat.p*.

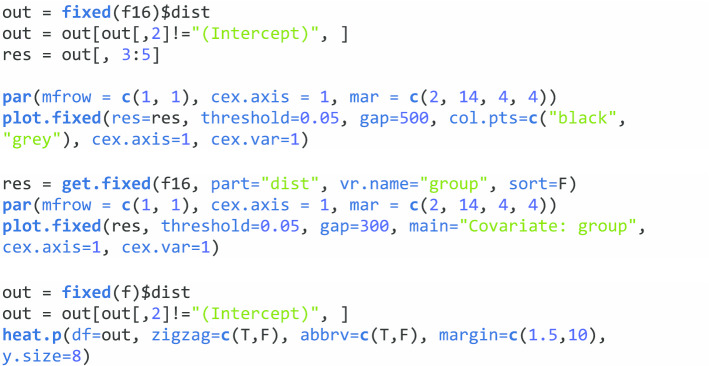


## Conclusions

We have developed a freely available R package **NBZIMM** to address some of the analytic challenges in complex microbiome/metagenomic studies. Although we emphasize microbiome/metagenomic data, the package and the methods are general and can be used to analyze other over-dispersed and zero-inflated count data with multilevel designs. **NBZIMM** package is under continual development. Our mixed models adopt a classical framework that is not appropriate to jointly analyze multiple correlated covariates. We will extend the mixed models by incorporating weakly informative prior distributions for the fixed effects that allow us to obtain more reliable and stable inferences [23]. We also plan to develop mixed models for jointly analyzing multiple taxa. Moreover, we found ZIGMMs had inflated false positive rate similarly as the R package **metagenomeSeq** [9]. Our plan for solution is to develop analyzing methods under Bayesian framework using MCMC algorithm to possibly address the current fitting issues.

## Availability and requirements

- Project name: NBZIMM
- Project home page: https://github.com/nyiuab/NBZIMM
- Operating system(s): Platform independent
- Programming language: R
- Other requirements: none
- License: MIT
- Any restrictions to use by non-academics: none

## Data Availability

The datasets used and analyzed during the current study are publicly available from Romero et al. [6] and Vincent et al. [21]. The package with manual is freely available from the public GitHub repository https://github.com/nyiuab/NBZIMM.

## Abbreviations

CDI: Clostridium difficile infected
EM: Expectation–maximization
EM-IWLS: Expectation–maximization-Iteratively reweighted least squares
NBZIMM: Negative binomial and zero-inflated mixed models
NBMMs: Negative binomial mixed models
OTU: Operational taxonomic units
ZIBR: Zero-inflated Beta regression
ZIGMMs: Zero-inflated Gaussian (linear) mixed models
ZINBMMs: Zero-inflated negative binomial mixed models
